# Caffeine Consumption Influences Lidocaine Action via Pain-Related Voltage-Gated Sodium Channels: An In Vivo Animal Study

**DOI:** 10.1155/2022/6107292

**Published:** 2022-01-04

**Authors:** Reham Alfaraj, Zainab Alabdulsalam, Zahrah Alfaraj, Hawraa Alsunni, Hussain Alhawaj, Omar Omar, Hatem Abuohashish

**Affiliations:** ^1^College of Dentistry, Imam Abdulrahman Bin Faisal University, Dammam 31441, Saudi Arabia; ^2^Institute for Research and Medical Consultations (IRMC), Imam Abdulrahman Bin Faisal University, Dammam 31441, Saudi Arabia; ^3^Department of Biomedical Dental Sciences, College of Dentistry, Imam Abdulrahman Bin Faisal University, Dammam 31441, Saudi Arabia

## Abstract

Several factors might influence the duration and efficiency of local anesthesia. This study investigates the effect of habitual caffeine intake on lidocaine action and explores the potential involvement of voltage-gated sodium channels in the interaction effect. Wistar rats were divided into four groups: (i) control (Ctrl), (ii) lidocaine intraplantar injection (LIDO), (iii) habitual caffeine intake (CAF), and (iv) lidocaine intraplantar injection and habitual caffeine intake (LIDO + CAF). Behavioral assessments, consisting of a paw pressure test for mechanical pressure sensation and a paw withdrawal latency test for thermal pain sensation, were performed at 0, 30, 60, and 90 minutes following lidocaine injection and after 10, 11, and 12 weeks of CAF intake. Pressure sensation was significantly reduced in the LIDO + CAF group compared with the control group. Moreover, the LIDO + CAF group exhibited reduced sensation compared to LIDO alone group. The LIDO + CAF combination exerted a synergistic effect at 30 and 60 minutes compared with the control. This synergistic effect was noted at 60 minutes (11 weeks of CAF intake) and at 30 minutes (12 weeks of CAF intake) compared with LIDO alone. Augmented thermal pain-relieving effects were observed in the LIDO + CAF group at all weeks compared to the control group and at 10 weeks compared to LIDO alone group. The molecular analysis of dorsal root ganglia suggested that CAF upregulated the mRNA expression of the Nav1.3, Nav1.7, and Nav1.8 sodium channel subtypes. Chronic caffeine consumption potentiates the local anesthetic action of lidocaine in an experimental animal model through mechanisms that involve the upregulation of voltage-gated sodium channels in the dorsal root ganglia.

## 1. Introduction

Lidocaine is a classical local anesthetic agent and belongs to the amino amide class of local anesthetics. Lidocaine is indicated to eliminate pain sensation and discomfort by inhibiting the propagation and generation of action potentials in peripheral neurons through the blockade of sodium channels. These channels are located in axonal neural membranes [1]. Nine sodium channels with approximately 50% similarity in the transmembrane segments and extracellular loop regions have been identified. The *α*-subunit of each channel identifies the members of sodium channels. These members are named Nav1.1 to Nav1.9 and have different expression patterns and physiological profiles. For example, voltage-gated sodium channel numbers 3 (Nav1.3), 7 (Nav1.7), and 8 (Nav1.8) are considered pain-related channels expressed in dorsal root ganglia [2].

Caffeine is a well-known natural compound present in various plant products. Caffeine is an integral component of several daily drinks, including tea, coffee, soft drinks, and energy drinks [3]. The consumption habits of these caffeinated drinks have facilitated the wide distribution of and dependence on caffeine. For instance, 89% of the US adult population has an average caffeine intake of 200 mg/day [4]. Among the multiple effects and side effects, caffeine also has an analgesic action and is used to reduce pain in several pharmaceutical forms as an adjuvant [3]. Several studies have reported a role for caffeine in controlling pain and suggested that caffeine may block the central processing of pain signals in the brain and enhance the body's natural pain resistance pathways [5]. In addition, Overstreet et al. found that habitual caffeine consumption diminishes pain sensitivity in a laboratory setting [6].

Clinically, dentists may encounter patients with reduced numbness effects following administration of local anesthesia and explain this because of excessive caffeine consumption. In this context, a cross-sectional study assumed that caffeine consumption could induce alertness and the resulting anxiety and stress might explain this incidence [7]. Moreover, the effects of chronic caffeine consumption on pain sensation and the duration of local anesthesia have not been scientifically elucidated. Therefore, the aim of the present study was to investigate the effect of chronic caffeine consumption on the effects and duration of action of lidocaine in an experimental animal model. In addition, a study exploring whether such an interaction effect is associated with altered mRNA expression of pain-related voltage-gated sodium channels was performed.

## 2. Materials and Methods

### 2.1. Experimental Animals and Ethical Considerations

Thirty-two female Wistar albino rats weighing approximately 250 grams were obtained from the animal house at Imam Abdulrahman Bin Faisal University (IAU). Animals were housed in standard polycarbonate cages (4 animals per cage) under measured pathogen- and stress-free laboratory conditions of 25°C and a 12-hour light/dark cycle. Animals had free access to a standard rat pellet diet (Grain Silos and Flour Mills Organization, Riyadh, Saudi Arabia). The general health was monitored thoroughly. Rats were provided free access to water and food throughout the experimental period. The experimental procedures performed in the present study followed the National Institute of Health (NIH) Guide for the Care and Use of Laboratory Animals (8th edition-2011). Moreover, this project was approved by the Animal Care and Use Committee (ACUC) at Imam Abdulrahman Bin Faisal University (IRB number 2019-137).

### 2.2. Animal Grouping and Study Design

**Figure 1:**
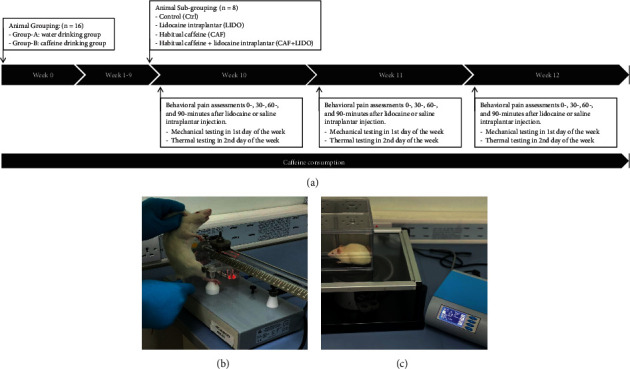
Experimental design and pain sensation testing. (a) Experimental design: at week 0, animals were allocated into group A, the water drinking group, and group B, the caffeine drinking group (*n* = 16). At the end of the 9th week, animals were subdivided into 4 subgroups (*n* = 8): control (Ctrl), receiving an intraplantar lidocaine injection (LIDO), habitual caffeine intake (CAF), and habitual caffeine intake and receiving an intraplantar lidocaine injection (CAF + LIDO). Mechanical testing was conducted on the 1st day of weeks 10, 11, and 12, while thermal testing was conducted on the 2nd day of weeks 10, 11, and 12. Both mechanical testing and thermal testing were performed 4 times per day (0, 30, 60, and 90 minutes) after intraplantar lidocaine or saline injections. (b) Mechanical pressure sensation testing (Randall–Selitto test): the tip of the Analgesy Meter was applied with increasing force on the dorsal surface of the rat's hind paw until a withdrawal response was obtained. (c) Thermal pain sensation testing (paw withdrawal latency test): an infrared light, a thermal heat stimulus, was placed underneath the injected paw until the withdrawal reflex was recorded.

Animals were initially divided into two main groups using block randomization (*n* = 16). Group A (water drinking group) drank tap water without caffeine, while group B (caffeine drinking group) drank tap water containing 300 mg/L caffeine [8]. By the end of the 9^th^ week of caffeine consumption, animals in each group were randomly subdivided into 2 subgroups (*n* = 8): a group administered an intraplantar saline injection and a group administered an intraplantar lidocaine injection. This division and subdivision resulted in a total of 4 experimental groups: (i) a control (Ctrl) group administered drinking water without caffeine and an intraplantar injection of saline; (ii) a lidocaine (LIDO) group administered drinking water without caffeine and an intraplantar injection of 0.25 mg of plain lidocaine; (iii) a caffeine (CAF) group administered drinking water with caffeine and an intraplantar injection of saline; (iv) a lidocaine plus caffeine (LIDO + CAF) group administered drinking water with caffeine and an intraplantar injection of 0.25 mg of plain lidocaine (Figure 1(a)). The intraplantar lidocaine injection was performed using a previously described procedure [9]. Lidocaine or saline was injected before each behavioral assessment. Animal cages were clearly labeled with the group code, animal numbers, and treatment doses. All authors were blinded to randomization and group allocation, except H Alhawaj and H Abuohashish. The sample size was calculated using G*∗*Power and based on an effect size estimated from previous studies on lidocaine [9] and caffeine [10]. The sample size was calculated for the repeated measures within-between interactions using the following criteria: effect size (*f*) of 0.25, alpha error probability (a) of 0.05, and power (1-b)) of 0.8.

### 2.3. Behavioral Testing Protocol

Behavioral assessments of mechanical and thermal pain sensations were performed once per week in the 10^th^, 11^th^, and 12^th^ weeks of caffeine consumption. The Randall–Selitto test (paw pressure test) was used to assess the mechanical pressure sensation on the first day of the week, while the paw withdrawal latency test was performed to evaluate thermal pain sensation on the second day of the week. Each behavioral test was repeated 4 times per day with an interval of 30 minutes (*t* = 0, 30, 60, and 90 minutes) following lidocaine or saline injections (Figure 1(a)). The behavioral pain assessments were conducted in a blind manner, where the identities of the tested animals were not revealed to the examiners. The same pattern of testing was applied until week 12, which was the last week of behavioral testing. All behavioral assessments were carried out in the animal house at Imam Abdulrahman Bin Faisal University.

### 2.4. Testing of Mechanical Pressure Sensation (Randall–Selitto Test)

This test was applied by increasing the force until the rat withdrew the paw using an Analgesy Meter hand-held device (model 37215, Ugo Basile S.R.L., 21036 Gemonio VA, Italy). Each animal was handled for five minutes to allow it to acclimate to the manipulation before the test, and a mark was generated at the location of application (plantar surface) to be maintained over repeated trials. The tip of the device was applied to the dorsal surface of the rat's hind paw until a withdrawal response was observed. A maximum of 250 g of force was applied to avoid skin damage (Figure 1(b)). Animals that exceeded the cutoff force were allowed to rest for 1 hour, and testing was repeated. Animals that repeatedly exceeded the cutoff force were excluded. Paw pressure tests were used to assess the nociceptive withdrawal threshold. The rats were assessed at 0, 30, 60, and 90 minutes after the intraplantar lidocaine injection and 3 weeks of caffeine consumption.

### 2.5. Testing of Thermal Pain Sensation (Paw Withdrawal Latency Test)

A paw withdrawal latency test was used to assess the heat threshold. The test was conducted by placing the animals, without restraint, on the surface of the original plantar test apparatus for thermal stimulation (Hargreaves Apparatus, model 37370, Ugo Basile S.R, L 21036 Gemonio VA, Italy). The thermal plantar stimulus (infrared light) was positioned underneath the rat's injected paw using a glass-bottom enclosure until withdrawal was observed. Paw withdrawal or licking, a leaning posture, stamping, and jumping were considered nocifensive behaviors (Figure 1(c)). The cutoff time was 60 seconds, and the time was recorded automatically. Animals exceeding the cutoff time were allowed to rest for 1 hour, and testing was repeated. Animals that repeatedly exceeded the cutoff time were excluded. The test was applied at all time intervals, *t* = 0, 30, 60, and 90 minutes, for 3 weeks starting from the 10^th^ week of caffeine consumption.

### 2.6. Sample Collection and Molecular Analysis

At the end of the 12th week, six randomly selected animals from each of the Ctrl and CAF groups (*n* = 6) were euthanized. Then, the spinal dorsal root ganglia of each animal were quickly dissected for PCR quantification of the mRNAs expressions of Nav1.3, Nav1.7, and Nav1.8. Briefly, an EZNA FFPE RNA Kit (R6954-00, Omega Biotek, Inc. 400 Pinnacle Way, Suite 450, Norcross, GA 30071, USA) was employed for total RNA isolation and purification from spinal dorsal root ganglia according to the manufacturer's protocol. Then, 1 *μ*g of the isolated RNA was reverse transcribed into single-stranded complementary DNA (cDNA) templates using the QuantiTect Reverse Transcription Kit (205311, Qiagen gmbh, Qiagen Strasse 1, 40724 Hilden, Germany), and random hexamer primers in a two-step RT-PCR. Real-time PCR was performed to evaluate the expression of the Nav1.3, Nav1.7, and Nav1.8 mRNAs using Rotor-Gene *Q* (Qiagen gmbh, Qiagen Strasse 1, 40724 Hilden, Germany), with *β*-actin serving as the housekeeping gene. The cDNA amplicons were amplified using Maxima SYBR Green/Fluorescein qPCR Master Mix (K0241 Thermo Scientific™, Waltham, MA, USA) with the specific primer sequences listed in Table 1 [11] according to the manufacturer's protocol. Values for the threshold cycle (Ct) were normalized to the average Ct value of the housekeeping gene (∆Ct). The fold changes were calculated as 2-∆∆ct.

### 2.7. Statistical Analysis

Results from the behavioral tests are presented as the means ± standard deviations. The effects of lidocaine and caffeine on pressure and thermal sensations were evaluated using a linear mixed model with repeated measures, with the testing time points (min) serving as the within-subjects factor, experimental groups (“control,” “LIDO,” “CAF,” and “LIDO + CAF”) serving as the between-subjects factor, and time by the experimental groups as the interaction term to evaluate how the effects of lidocaine and caffeine or their combination are influenced by the time of testing. The data were analyzed after splitting into weekly intervals (10, 11, and 12 weeks) of caffeine administration. In the linear mixed model, the main effects and the time-specific differences between the groups were compared with adjustment using the Bonferroni test. All statistical analyses were conducted using IBM® SPSS® version 25. *P* values < 0.05 were considered statistically significant. The results of the PCR analysis are presented as the means ± standard deviations and were statistically analyzed using two-tailed paired *t*-tests. Significance was considered when the *p* values were less than 0.05. Statistical analyses were performed using GraphPad Prism (version 5) software. A grayscale heat map was generated using an online heatmapper [12].

## 3. Results

### 3.1. Effects on Mechanical Pressure Sensation

**Figure 2:**
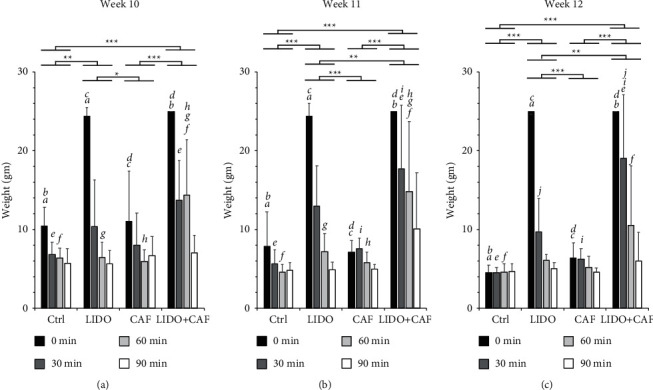
Effects on the mechanical pressure sensation test. The bar graph shows the weights (gm) applied to the rat limb until retraction and were tested 0, 30, 60, and 90 minutes after the local (intraplantar) administration of lidocaine. The rats were divided into 4 subgroups (*n* = 8) as follows: control (Ctrl), receiving a local lidocaine injection (LIDO), habitual caffeine intake (CAF), and habitual caffeine intake and receiving a local lidocaine injection (CAF + LIDO). Caffeine was provided in the drinking water to the respective groups for 10-week (a), 11-week (b), and 12-week (c) periods. The statistical analysis was performed using a linear mixed model with repeated measures, and the main effects and time-specific differences were compared with adjustment using the Bonferroni test. Significant differences between the experimental groups are shown with the connecting bars and asterisks (^*∗*^ < 0.05, ^*∗∗*^ < 0.01,^*∗∗∗*^ < 0.0001). Significant differences between the groups for the individual time points are indicated with small letters, where every two similar letters indicate a statistically significant difference (*p* < 0.05) between the two groups they represent for the specific time point.

For pressure sensation, the linear mixed model analysis with repeated measures revealed a significant fixed effect of LIDO alone compared with the control at all weekly intervals (i.e., 10, 11, and 12 weeks) (Figures 2(a)–2(c)). In contrast, no significant fixed effect was observed for the animals treated with CAF alone or the control group at any of the weekly intervals of caffeine intake. A statistically significant effect (*p* < 0.001) was observed for the combination group (LIDO + CAF) at each evaluation week compared to the control group (Figures 2(a)–2(c)). In addition, the effect of the combination group (LIDO + CAF) was significantly higher than that of LIDO alone, but mainly after the 11th and 12th weeks (*p* < 0.01) of CAF intake. When considering the duration of the effect (in minutes), the significant effect of LIDO compared with the control was mainly detected at 0 minutes after testing, but not after 30, 60, or 90 minutes in the 10th, 11th, and 12th weeks (Figures 2(a)–2(c)). On the other hand, the combination group (LIDO + CAF) exhibited significant effects compared to the control at longer durations (30 and 60 minutes) in the 11th and 12th weeks of caffeine intake. In fact, the combination group (LIDO + CAF) displayed a significant effect at 60 minutes compared to LIDO alone, particularly at the 11-week interval (Figures 2(a) and 2(b)). Nonetheless, at the longer period of caffeine intake (12 weeks), the synergistically increased effect observed in the combination group (LIDO + CAF) compared with LIDO alone was limited to the 0- and 30-minute time points during testing (Figure 2(c)). No significant differences in pressure test results were observed at 90 minutes between any of the experimental groups.

### 3.2. Effects on Thermal Pain Sensation

**Figure 3:**
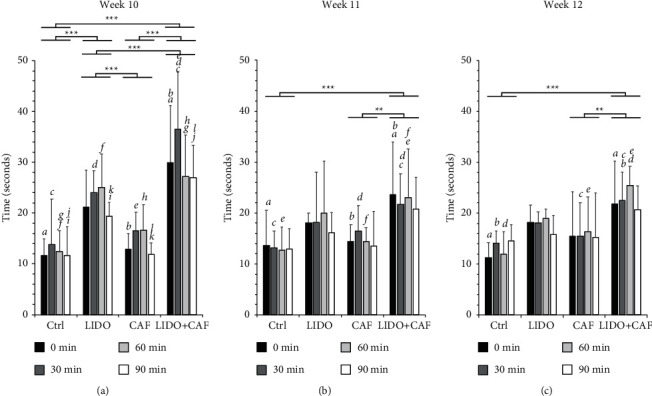
Effects on thermal pain sensation test. The bar graph shows the time (seconds) from the application of the heat stimulus until the rat retracted its paw and was tested 0, 30, 60, and 90 minutes after the local (intraplantar) administration of lidocaine. The rats were divided into 4 subgroups (*n* = 8) as follows: control (Ctrl), receiving a local lidocaine injection (LIDO), habitual caffeine intake (CAF), and habitual caffeine intake and receiving a local lidocaine injection (CAF + LIDO). Caffeine was provided in the drinking water to the respective groups for 10-week (a), 11-week (b), and 12-week (c) periods. The statistical analysis was performed using a linear mixed model with repeated measures, and the main effects and time-specific differences were compared with adjustment using the Bonferroni test. Significant differences between the experimental groups are shown with the connecting bars and asterisks (^*∗*^ < 0.05, ^*∗∗*^ < 0.01,^*∗∗∗*^ < 0.0001). Significant differences between the groups for the individual time points are indicated with small letters, where every two similar letters indicate a statistically significant difference (*P* < 0.05) between the two groups they represent for the specific time point.

For the thermal sensation, the linear mixed model analysis with repeated measures revealed a significant fixed effect (*p* < 0.001) of LIDO alone compared with the control, but only at the 10-week interval (Figure 3(a)). Furthermore, at the 10-week interval of caffeine intake, a significantly greater effect was observed on the combination group (LIDO + CAF) compared with the control (*p* < 0.001), LIDO alone (*p* < 0.001), and CAF alone (*p* < 0.001) groups. At the latter intervals of caffeine intake, a significantly greater effect was observed on the combination group (LIDO + CAF) compared with the control and CAF groups, but not compared with the group treated with LIDO alone (Figures 3(b) and 3(c)). In addition, the significant increase in heat perception observed in the combination group (LIDO + CAF) was extended up to 90 minutes (for the 10-week interval of CAF intake) (Figure 3(a)) and up to 60 minutes (for the 11- and 12-week intervals of CAF intake) (Figures 3(b) and 3(c)).

### 3.3. Effects on Voltage-Gated Sodium Channels Expressions

**Figure 4:**
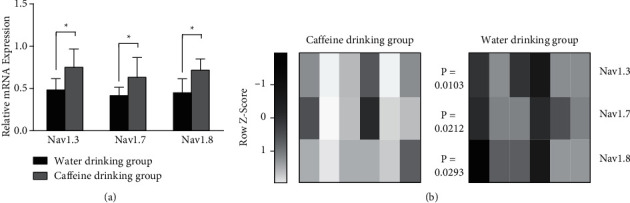
Expression of pain-related voltage-gated sodium channel genes. (a) Relative mRNA expression of pain-related voltage-gated sodium channels, including the Nav1.3, Nav1.7, and Nav1.8. (b) Grayscale heat map generated using the online heatmapper [12] showing the distribution of Nav1.3, Nav1.7, and Nav1.8 mRNA expression levels as Z-scores for each animal in the water drinking group and caffeine drinking group. *P* values of the two-tailed paired *t*-test are reported. Differences were considered significant for *p* values < 0.05 (^*∗*^).

In the dorsal root ganglion, chronic caffeine consumption for 12 weeks significantly (*P* < 0.05) upregulated the expression of pain-related voltage-gated sodium channels, including the Nav1.3, Nav1.7, and Nav1.8 mRNAs (Figure 4(a)). The distribution of Nav1.3, Nav1.7, and Nav1.8 relative mRNA expression levels among the water drinking group and caffeine drinking group is presented in Figure 4(b). The Z-score for every single animal in each group showed a homogenous distribution, with a higher score recorded for the caffeine drinking group than in the water drinking group (Figure 4(b)).

## 4. Discussion

Chronic caffeine intake is a global routine in the form of drinks, foods, and medications. This habitual intake influences physiological pathways and hence the pharmacokinetic and pharmacodynamic properties of several medications. The present study investigated whether chronic caffeine consumption might alter the efficacy of a commonly used local anesthetic agent (lidocaine) in experimental animals. Caffeine was daily administered in the drinking water at a low concentration of 300 mg/L (approximately 3 mg/kg/day), which is comparable to the average intake (200 mg/day) [4] of an adult human (70 kg). Pain assessments were conducted by measuring the mechanical and thermal sensations after the application of pressure or heat stimuli that aggravate pain [13]. The findings from the present study revealed that the effects of lidocaine might be influenced by chronic caffeine intake. The improvement in the response to mechanical pressure stimulation was more prominent. In addition, the effect of caffeine consumption on lidocaine action was associated with changes in the mRNA expression of pain-related voltage-gated sodium channels such as Nav1.3, Nav1.7, and Nav1.8 in the dorsal root ganglia, which are the main target of lidocaine actions.

In the current study, the baseline withdrawal thresholds and latencies were not significantly altered by chronic caffeine intake at different time points compared to the control group. Based on these results, caffeine does not exert a local anesthetic effect itself after systemic administration for 12 weeks. This result is consistent with the variable findings from previous studies that described the effects of caffeine on thermal and mechanical sensations. Caffeine reduced mechanical allodynia in carrageenan-stimulated mice [14]. Caffeine also exerted short-term antinociceptive effects on the paw withdrawal latency after exposure to a noxious thermal stimulus [10]. In the study by Galeotti et al. [15], caffeine restored morphine withdrawal-induced hyperalgesia, which was associated with an increased pain threshold in mice with hyperalgesia.

Animals without caffeine consumption showed significantly higher withdrawal thresholds and delayed latencies after local lidocaine administration at assessments performed in different weeks. Notably, local anesthetic effects of lidocaine on mechanical and thermal sensations did not last for 30, 60, or 90 minutes after intraplantar injection. Similar results were reported in the study by Shao et al. [16], where the intraplantar injection of lidocaine alone resulted in short-term (45 min), substantial increases in the paw withdrawal latency and mechanical withdrawal threshold. In addition, lidocaine showed short-lived efficacy against thermal and mechanical hyperalgesia after an intraplantar injection in diabetic and nondiabetic rats [17]. Lidocaine is the most widely employed local anesthetic agent in dentistry. The duration of action of a pulpal lidocaine injection lasted for approximately 40 minutes in the study by Costa et al. [18] and 60 minutes in the study by Tortamano et al. [19]. These studies used lidocaine in combination with 1 : 100,000 epinephrine. However, in our study, we tested the effects of plain lidocaine and its duration to avoid interference from the vasoconstrictive effects of epinephrine. The duration of plain lidocaine action in dentistry is short. Therefore, it is usually combined with vasoconstrictor agents such as epinephrine. In our study, the duration of action of plain lidocaine was similar to its duration of action when used without epinephrine in dentistry.

Lidocaine action in animals consuming caffeine was compared with animals without caffeine consumption, including withdrawal thresholds and latencies after the intraplantar lidocaine injection. Caffeine intake influenced the lidocaine anesthetic action, particularly against mechanical pressure pain sensation, in assessments performed in all weeks. This synergistic effect lasted up to 60 minutes after the lidocaine injection. Notably, the significance of differences between the control group and animals receiving combined treatments was higher than the significance of differences between the control group and animals treated with lidocaine alone. Meanwhile, the thermal latencies were delayed in caffeine-treated animals after lidocaine administration for up to 60 minutes at the 11^th^ and 12^th^ weeks and up to 90 minutes at the 10^th^ week. Based on these findings, the duration of chronic caffeine consumption influences the regional anesthetic action of lidocaine. In the study by Reynolds et al. [20], average daily caffeine intake had no relationship with pain sensation in subjects subcutaneously injected with or without a lidocaine solution, which differs from our results. Premnath et al. [7] suggested that caffeine consumption causes a failure of local anesthesia by inducing alertness and anxiety. Our findings are not consistent with this explanation because caffeine consumption enhanced the pain threshold, particularly the threshold for mechanical pain sensation, after lidocaine injection. Scientific pharmacological elucidation of our outcomes was provided by assessing the effect of caffeine on the expression of voltage-gated sodium channels, which are the intracellular target of lidocaine action.

Our results were also confirmed by measuring the effect of caffeine on voltage-gated sodium channels. Our study is the first to investigate the effect of caffeine consumption on pain-related voltage-gated sodium channels in the dorsal root ganglion. Voltage-gated sodium channels are the main target of lidocaine action, leading to the blockade of sodium ion influx and action potentials. Nav1.3, Nav1.7, and Nav1.8 are pain-related channels that are mainly expressed in dorsal root ganglia [2]. These channels are more closely related to mechanical pain sensation than thermal pain sensation [13]. The upregulated mRNA expression of pain-related Nav1.3, Nav1.7, and Nav1.8 channels suggested that caffeine consumption might increase the abundance of lidocaine targets, which increases their efficacy, predominantly against mechanical pain sensation. The relationship between caffeine and sodium channels was documented previously but not in relation to pain sensation. Zhao et al. [21] studied the effect of caffeine on voltage-dependent currents and found that caffeine produced weak inhibition of the sodium current in rat taste receptor cells. Similar findings were reported in other studies, where caffeine reduced sodium currents in guinea pig single ventricular cells [22] and in mouse atria [23]. Sarbjit-Singh et al. [24] found that caffeine may inactivate the Nav1.4 channel in murine skeletal muscle fibers, leading to sodium current inhibition. Moreover, levels of the Nav1.5 and NaX proteins were downregulated by caffeine, changes that were associated with increased intracellular calcium concentrations in cardiac myocytes [25]. On the other hand, the main transducer in thermal (heat) pain sensory neurons is transient receptor potential cation channel subfamily V member 1 (TRPV1), where temperature gradually increases its activity [13]. In the study by Eberhardt et al. [26], lidocaine action on the dorsal root ganglion did not depend on ion influx through TRPV1. Furthermore, blockade of TRPV1 resulted in a reduced effect of caffeine on blood vessels [27]. These reports might explain why the synergistic effects of lidocaine and caffeine were reported mainly on mechanical pain, where Nav channels play a vital role. However, in the case of thermal pain sensation, lidocaine action might not require TRPV1. Further studies are required to explore the effect of caffeine consumption on TRPV1 expression and sensitivity in the dorsal root ganglion.

Two limitations were encountered in this study. First, the lidocaine used in this study was plain; hence, the effect of epinephrine was not considered. Since the main objective of the study was to determine the effect of caffeine on lidocaine action, other potential influencing factors were removed. The second limitation is that behavioral testing should have been conducted after 120 minutes to better understand the effect of caffeine on the lidocaine duration of action. However, behavioral testing was limited to 90 minutes to avoid animal stress, which might produce variabilities and discrepancies in results.

## 5. Conclusions

Taken together, our findings indicate that chronic caffeine consumption enhances the local effects of lidocaine. One novel finding of this study was the effect of caffeine on pain-related voltage-gated sodium channel expression in the dorsal root ganglion, which explains the enhanced lidocaine efficacy induced by caffeine consumption. The findings of this study are worthy of validation in further clinical investigations.

## Figures and Tables

**Table 1:** Primer sequences of sodium channel alpha subunit genes according to NCBI gene database.

Gene name	Official abbreviation	Accession number	Sequences
Sodium voltage-gated channel alpha subunit 3	Scn3a (Nav1.3)	NM_013119.2	CGGCTCAAAGAAACCTCAGA
			TCGAGAGAATCACCACCACA

Sodium voltage-gated channel alpha subunit 9	Scn9a (Nav1.7)	NM_133289.2	TTCGGCTCATTCTTCACGTT
			CACTCCCCAGTGAACAGGAT

Sodium voltage-gated channel alpha subunit 10	Scn10a (Nav1.8)	NM_017247.2	CACGGATGACAACAGGTCAC
			GATCCCGTCAGGAAATGAGA

## Data Availability

All data supporting the findings of this study are available within the manuscript.
